# Plasma microglial-derived extracellular vesicles are increased in frail patients with Mild Cognitive Impairment and exert a neurotoxic effect

**DOI:** 10.1007/s11357-023-00746-0

**Published:** 2023-02-01

**Authors:** C. Visconte, M.T. Golia, C. Fenoglio, M. Serpente, M. Gabrielli, M. Arcaro, F. Sorrentino, M. Busnelli, A. Arighi, G. Fumagalli, E. Rotondo, P. Rossi, B. Arosio, E. Scarpini, C. Verderio, D. Galimberti

**Affiliations:** 1 Department of Biomedical, Surgical and Dental Sciences, University of Milan, Milan, Italy; 2 CNR, Institute of Neuroscience, Vedano al Lambro, Monza and Brianza Milan, Italy; 3 Department of Physiopathology and Transplantation, University of Milan, “Dino Ferrari” Center, Milan, Italy; 4 Fondazione, IRCCS Ca’ Granda, Ospedale Maggiore Policlinico, Milan, Italy; 5 Department of Clinical Sciences and Community Health, University of Milan, Milan, Italy

**Keywords:** Extracellular vesicles, Frailty, Mild cognitive impairment, Microglial derived extracellular vesicles, Neuronal derived extracellular vesicles

## Abstract

Extracellular vesicles (EVs) are mediators of cellular communication that can be released by almost all cell types in both physiological and pathological conditions and are present in most biological fluids. Such characteristics make them attractive in the research of biomarkers for age-related pathological conditions. Based on this, the aim of the present study was to examine the changes in EV concentration and size in the context of frailty, a geriatric syndrome associated with a progressive physical and cognitive decline. Specifically, total EVs and neural and microglial-derived EVs (NDVs and MDVs respectively) were investigated in plasma of frail and non-frail controls (CTRL), mild cognitive impairment (MCI) subjects, and in Alzheimer’s disease (AD) patients. Results provided evidence that AD patients displayed diminished NDV concentration (3.61 × 10^9^ ± 1.92 × 10^9^ vs 7.16 × 10^9^ ± 4.3 × 10^9^ particles/ml) and showed high diagnostic performance. They are able to discriminate between AD and CTRL with an area under the curve of 0.80, a sensitivity of 78.95% and a specificity of 85.7%, considering the cut-off of 5.27 × 10^9^ particles/ml. Importantly, we also found that MDV concentration was increased in frail MCI patients compared to CTRL (5.89 × 10^9^ ± 3.98 × 10^9^ vs 3.16 × 10^9^ ± 3.04 × 10^9^ particles/ml, *P* < 0.05) and showed high neurotoxic effect on neurons. MDV concentration discriminate frail MCI vs non-frail CTRL (AUC = 0.76) with a sensitivity of 80% and a specificity of 70%, considering the cut-off of 2.69 × 10^9^ particles/ml. Altogether, these results demonstrated an alteration in NDV and MDV release during cognitive decline, providing important insight into the role of EVs in frailty status.

**Supplementary Information:**

The online version contains supplementary material available at 10.1007/s11357-023-00746-0.

## Introduction

Extracellular vesicles (EVs) are a heterogeneous group of vesicles with a size ranging from 50 to 500 nm, which can be released by almost all cell types both in physiological and pathological conditions, and are present in most biological fluids [1]. EVs are mediators of cellular communication, able to transfer part of their “cargo” to other cells, including proteins, lipids and nucleic acids in the form of DNA, mRNA transcripts, non-coding (nc)RNAs (long ncRNAs, microRNA and circular RNA) [2, 3]. Accumulating evidence suggests that EVs have a role in the functioning of the central nervous system (CNS), providing a mechanism of communication between nerve and glial cells, as well as between the CNS and all body systems, since EVs can cross the blood-brain barrier (BBB) [4].

Although the exact mechanism of EVs efflux remains to be clarified, a few hypotheses have been already postulated, including the direct translocation into capillaries or transportation into the perinasal lymphatics and then into the venous system [5].

Moreover, EVs have emerged to exert important roles in neurodegenerative diseases [6]. Notably, several studies have investigated the content of plasma vesicles of neuronal and astrocytic origin involved in pathological processes associated with Alzheimer’s disease (AD), a chronic progressive neurodegenerative disorder characterized by devastating cognitive and memory decline [7, 8].

AD represents the most common cause of dementia in the elderly, and its prevalence has been calculated to rise in the next decade (https://www.alzforum.org/). The major histopathological hallmarks of AD are extracellular senile plaques and intracellular neurofibrillary tangles mainly in cortical and hippocampal regions. Senile plaques are characterized by abnormal accumulation of amyloid Aβ-peptide (Aβ) that can spontaneously self-aggregate into multiple coexisting physical forms, whereas neurofibrillary tangles result from the self-association of hyperphosphorylated microtubule-associated protein tau. Most patients who receive a specific diagnosis of AD dementia are unfortunately diagnosed when they are already progressed to the moderate or severe stages of disease [9]. Mild cognitive impairment (MCI) represents a transitional state that can precede dementia, characterized by cognitive decline that is not associated with any significant functional disability [10, 11].

Recently, a growing body of epidemiological evidence suggests connections between frailty, cognitive impairment and dementia [12, 13]. The term frailty refers to a geriatric syndrome characterized by progressive physical decline, an increase in an individual’s vulnerability to endogenous and exogenous stressors, leading to augmented dependency and/or death [14].

It has been observed that frailty increases the risk of cognitive decline, and that cognitive decline, in turn, may increase the risk of frailty. Mutual influences between frailty and impaired cognition have led to the definition of “cognitive frailty” [13, 15, 16], a pathological status characterized by a combination of MCI and frailty. This term is used to define a state of cognitive decline that is not accompanied by any significant functional disability [17, 18] and that often represent the prodromal AD phase.

Interestingly, EVs were reported to carry Aβ peptides, tau proteins and many other molecules relevant to AD pathology [19–21]. In addition, several studies suggest the use of specific vesicle subpopulations as prognostic biomarkers for neurological pathologies and diseases associated with aging [22, 23]. Although previous studies demonstrated that EV concentration decreases with advancing age [24], the relevance of EVs in the pathophysiology of frailty still remains unknown and needs to be explored.

In addition, recent studies demonstrate an involvement of specific brain tissue–derived EVs in AD. In particular, miRNA cargo in plasma neuronal-derived vesicles, NDVs, has been associated with AD [25]. Moreover, EVs from microglia, MDVs, have been found at high level in dementia patients [26] .

Among many aspects of frailty that have not been elucidated so far, there is the involvement of EVs in the disease pathophysiology as well as their possible exploitation as early disease biomarker. In the present study, we examined the relationship between the frailty status and the concentration of total plasma EVs from subjects with or without MCI and AD patients. In addition, we measured NDVs and MDVs to investigate the possible link between frailty and/or cognitive decline and size and concentration of brain-derived EV subtypes.

## Materials and methods

### Study population and sample collection

For the present study, 20 MCI, 20 AD patients and 20 controls (CTRL) were enrolled. Patients were recruited at the Alzheimer Center of the University of Milan, Fondazione Ca’ Granda, IRCCS Ospedale Maggiore Policlinico. After the diagnostic work-up, subjects were diagnosed by expert neurologists with MCI or AD, according to the specific criteria of each syndrome [27, 28]. Specifically, AD and MCI patients underwent a clinical interview, neurological and neuropsychological examination, routine blood tests, brain MRI and lumbar puncture (LP) for quantification of the cerebrospinal fluid (CSF) biomarkers Aβ-amyloid 42, total tau and tau phosphorylated at position 181 (Ptau181) as previously described [29]. The control group consisted of 20 non-demented volunteers matched for ethnic background and age and without memory and psycho-behavioral dysfunctions (MMSE ≥ 28). All participants of this study were further classified as frail and non-frail, according to Canevelli’s Frailty Index (FI), which defines frailty as a product of a cumulative deficits’ model that could be considered as an objective marker of “biological aging” [30].

It consists of 50 items in a checklist of non-predefined variables constituted by symptoms, signs, diseases, disabilities and laboratory findings. The FI is the ratio between deficits presented by the individual and the total number of deficits considered. The median of all frailty indices (0.15 ± 0.03) was used as a cutoff to define frail and non-frail subjects.

Informed consent to participate in this study was given by all subjects or their caregivers. The study was approved by the local ethical committee (Parere 532_2019bis del 13-6-2019 — by Comitato Etico Milano Area 2).

### Plasma collection

EDTA-blood was withdrawn at the time of diagnosis and was allowed to sit at room temperature for a minimum of 30 min and a max of 2 h. Plasma was isolated by centrifugation at 2500×g for 15 min at room temperature, divided in 500-μl aliquots and stored at − 80 °C until use. Details of the clinical and biological variables of patients and CTRL are reported in Table 1.

**Table 1:** Clinical and biological variables of patients and controls. *AD* Alzheimer disease, *MCI* mild cognitive impairment, *CTRL* controls, *MMSE* Mini-Mental State Examination score, *NDVs* neuronal-derived vesicles, *MDVs* microglial-derived vesicles

Variable	**AD**	**Frail AD**	**Non-frail AD**	**Frail MCI**	**Non-frail MCI**	**Frail CTRL**	**Non-frail CTRL**	***p-*value**
*N*	20	10	10	10	10	10	10	
Gender (M/F)	9:11	4:6	5:5	6:4	6:4	4:6	4:6	
Mean Aβ_42_ ± SEM (pg/ml)	446.5 ± 1.226	478.8 ± 1.99	414.2 ± 1.307	817.7 ± 3.9	847.6 ± 3.86	**–**	**–**	
Mean h-Tau ± SEM (pg/ml)	769.65 ± 4.012	703.8 ± 4.988	835.5 ± 6.36	452.9 ± 5.37	450.6 ± 5.38	**–**	**–**	
Mean *p*-Tau ± SEM (pg/ml)	87.325 ± 0.828	77.05 ± 0.939	97.6 ± 1.286	79.83 ± 2.33	73.29 ± 1.94	**–**	**–**	
Mean age, years ± SD	72.10 ± 7.64	73.857 ± 4.74	72.8 ± 7.75	75.1 ± 5.97	72.2 ± 5.7	81.5 ± 4.55	75.5 ± 5.52	
MMSE	18.21	14	22.8	26.3	26.4	28.2	28.6	
Mean Frailty Index (FI) ± SEM	0.145 ± 0.052	0.213 ± 0.046	0.0.074 ± 0.03	0.212 ± 0.039	0.057 ± 0.053	0.38 ± 0.034	0.083 ± 0.031	
Total EV concentration (EVs/ml)	7.24 × 10^10^ ± 5.02 × 10^10^	5.69 × 10^10^ ± 5.16 × 10^10^	8.68 × 10^10^ ± 4.63 × 10^10^	9.39 × 10^10^ ± 6.34 × 10^10^	5.87 × 10^10^ ± 4.72 × 10^10^	6.90 ×10^10^ ± 5.40 × 10^10^	4.48 × 10^10^ ± 2.80 × 10^10^	
Total EV size (nm)	138.89 ± 18.63	140.16 ± 23.42	137.61 ± 13.44	135.84 ± 13.26	149.01 ± 13.89	151.31 ± 24.2	145.14 ± 23.71	
NDV concentration (EVs/ml)	**3.61 × 10**^**9**^ **± 1.92 × 10**^**9**^	**3.65 × 10**^**9**^ **± 1.98 × 10**^**9**^	**3.58 × 10**^**9**^ **± 1.98 × 10**^**9**^	5.37 × 10^9^ ± 3.36 × 10^9^	5.18 × 10^9^ ± 3.36 × 10^9^	4.73 × 10^9^ ± 4.23 × 10^9^	**7.16 × 10**^**9**^ **± 4.3 × 10**^**9**^	***p*** < 0.05*
NDV size (nm)	139.81 ± 20.99	140.32 ± 24.95	139.35 ± 18.09	145.38 ± 39.9	146.6 ± 27.9	143.34 ± 27.02	159.66 ± 34.12	
MDV concentration (EVs/ml)	4.21 × 10^9^ ± 3.49 × 10^9^	4.08 × 10^9^ ± 3.55 × 10^9^	4.31 × 10^9^ ± 3.63 × 10^9^	**5.89 × 10**^**9**^ **± 3.98 × 10**^**9**^	4.88 × 10^9^ ± 3.43 × 10^9^	5.00 × 10^9^ ± 4.97 × 10^9^	**3.16 × 10**^**9**^ **± 3.04 × 10**^**9**^	***p*** < 0.05*
MDV size (nm)	150.04 ± 34.49	136.20 ± 19.04	161.12 ± 40.72	138.02 ± 20.68	153.56 ± 22.98	155.97 ± 32.27	153.7 ± 29.56	

### Isolation and purification of total, neuronal and microglial EVs from plasma

Total EV isolation protocol established that 2.5-μl thrombin (System Bioscience, Palo Alto, CA, United States) was added to 250-μl aliquots of plasma to induce clot formation and allow the removal of fibrin and related proteins. Reactions were mixed by inversion, incubated 5 min at room temperature, and then centrifuged at 10.000×g for 5 min. Thereafter, the supernatants were transferred into new tubes, and 67 μl of Exoquick® exosome precipitation solution (system Bioscences Inc., Mountainview, CA) was added, gently mixed by inversion and let to stand 30 min at 4 °C and further 30 min on ice. Next, suspensions were centrifuged at 3000×g for 10 min to obtain pellets containing total EVs. Supernatants were collected and stored (DEVs- depleted EVs), and pellets were re-suspended in 400 μl of Buffer B and Buffer A of ExoQuick Ultra at ratio of 1:1. The EV suspension was loaded to a pre-cleaned resin column with other 100 μl of Buffer B. After mixing, purified EVs were collected by centrifugation at 1000×g for 2 min. All separated EVs were aliquoted and stored at − 80 °C.

To enrich for neuronal and microglial EVs, anti-human CD171 (L1 Cell Adhesion Molecule-L1CAM) biotinylated antibody (clone 5G3, e Bioscience, San Diego, CA) or TMEM119 (Transmembrane Protein 119) extracellular biotinylated antibody (Biolegend, San Diego, CA, USA) was used, respectively; specifically, total EVs were incubated with 4 μg of anti CD171 or TMEM119 antibodies in 50 μl of 3% BSA for 4 h at 4 °C with continuous gentle mixing on a rotational mixer.

**Fig. 1:**
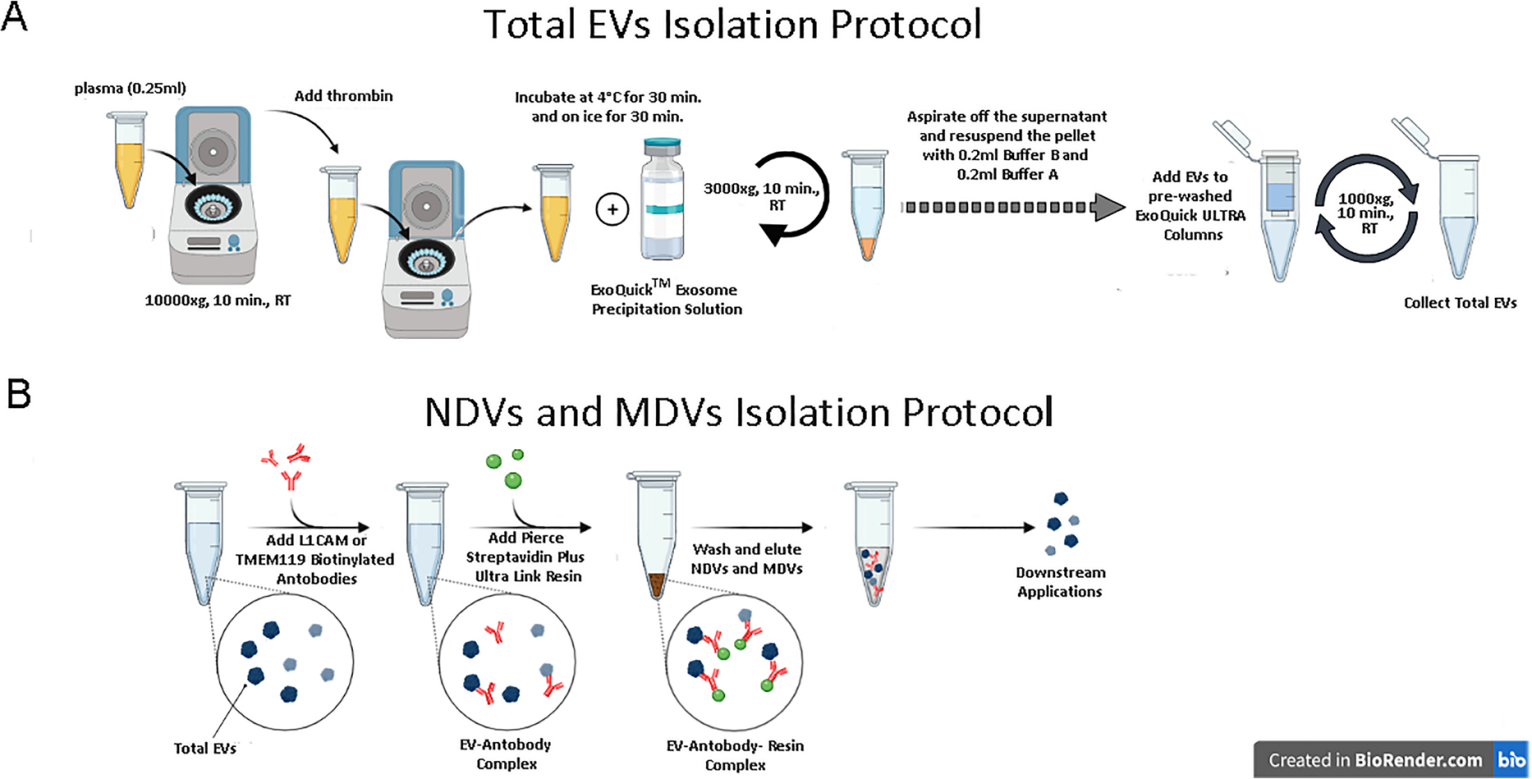
Plasma brain–derived extracellular vesicles can be isolated from peripheral blood, flowchart of the isolation protocol

Next, suspensions received 15 μl of streptavidin-agarose Ultralink resin (Thermo Scientific, Rockford, IL, USA) and 25 μl of 3% BSA and were further incubated for 60 min at 4 °C on a rotational mixer with continuous gentle mixing. Pellets, re-suspended in 200 μl of 0.1 M glycine-CTRLl solutions (pH 3.0), were vigorously mixed for 10 s and centrifuged at 4500×g for 5 min at 4 °C to detach L1CAM^+^ EVs and TMEM119^+^ EVs from the bead-antibody complex. Supernatants containing L1CAM^+^ EVs or TMEM119^+^ EVs were transferred to clean tubes containing 15 μl of 1 M TRIS-CTRLl pH 8.0 (to neutralize the pH) and mixed. Final suspensions of NDVs and MDVs were stored at − 80 °C until immediately prior to assays[7, 31]. A workflow of the complete protocol is shown in Fig. 1.

### Characterization of EVs by NTA

The size distribution and concentration of total, neuronal and microglial EVs were measured with NanoSight (NS300) (Malvern Instruments Ltd., Malvern, UK). To gain an optimal number of particles per field of view, EV suspensions were diluted with 0.1-μm filtered PBS, and four videos of 30 s in duplicate were recorded. Relative data were analysed under constant setting using Nanoparticle tracking Analysis (NTA) software 3.2 (Malvern Instruments Ltd., Malvern, UK) based on manufacturer’s recommendations. To assess the quality of the analysis, the ratio between the total particle tracks and the valid particle tracks was considered for each sample; the analysis was considered valid when the ratio was less than 5. The quantification of EVs is described per millilitre. NTA analysis was also used to provide the mean and mode of EVs.

### Characterization of EVs by Immunoblotting

To lyse NDVs and MDVs, each tube received 1 volume of M-PER mammalian protein extraction reagent (Thermo Scientific, Rockford, IL, USA), containing protease and phosphatase inhibitors (Thermo Scientific, Inc.).

**Fig. 2:**
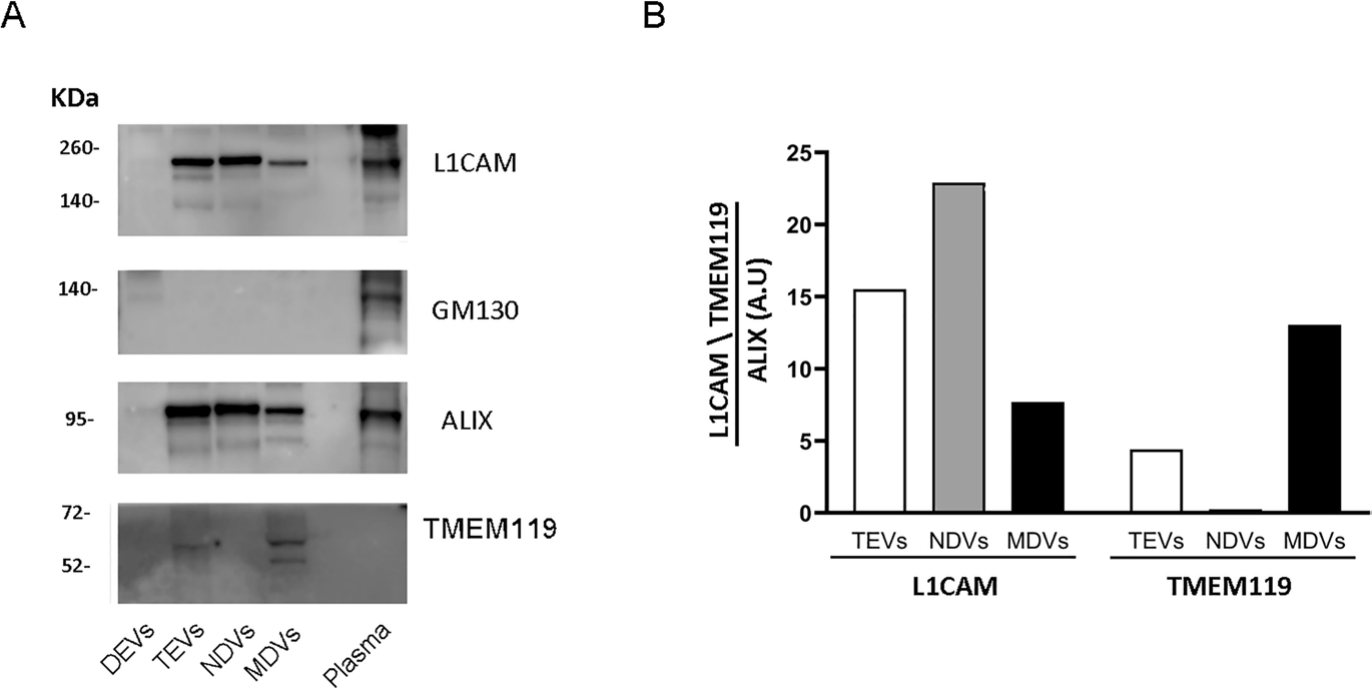
Characterization of total EVs (TEVs), NDVs and MDVs through Western Blot. **A** Representative expression of neuronal marker L1CAM, microglial marker TMEM119, vesicular markers ALIX and Golgi marker GM130 in TEV and EV subpopulations. Plasma sample and EV depleted fraction (DEVs) were used as positive and negative controls, respectively. **B** Relative quantification of L1CAM and TMEM119 in TEVs, NDVs and MDVs. L1CAM and TMEM119 expression was normalized to ALIX staining

Aliquots of the same amount of EVs lysates were separated by SDS-PAGE, on 4–12% acrylamide gels, and proteins were transferred to PVDF membrane. After blocking for 2 h with 5% BSA in TBS, membranes were incubated overnight at 4 °C with the desired primary antibodies diluted in 5% BSA in TBS. In the present study, the following antibodies and dilution were used: anti L1CAM 1:200 (ab24345, Abcam, Cambridge, MA, USA), anti TMEM119 1:500 (BioLegend, San Diego, CA, USA), anti ALIX 1:500 (1A12-sc53540, Santa Cruz Biotechnology, CA, USA), anti GOLGI2/GM130 1:500 (FNab03558, FineTest). Membranes were washed, incubated with appropriate peroxidase-conjugated secondary antibody (1:10000 dilution), and proteins were visualized with a chemiluminescence reaction, using the Odyssey Fc imager (LI COR). Quantification of band intensity was performed by computer-assisted densitometric scanning using ImageJ. The blots reported in Fig. 2 are representative images of at least three different experiments.

### Cytotoxicity assay

Neuron viability was analysed by simultaneous fluorescence staining of viable and dead cells with calcein-AM (0.5 mg/ml, Invitrogen, Life Technologies Ltd.) and propidium iodide (PI) (1 μg/ml, Molecular Probes, Life Technologies Ltd., Paisley, UK), as previously described [26]. Calcein-AM emits signal in viable neurons while PI binds to nuclei of dead cells only. Murine hippocampal neurons were plated on 24 multiwell (100 K cells/well) and cultured in neuronal medium (500 μl) for 10–11 days in vitro. Neurons were then exposed to two different concentrations of NDVs/MDVs for 24 h at 37 °C and 5% CO_2_. Briefly frozen aliquots of NDVs or MDVs+ EVs isolated from 100 μl of plasma were first washed in PBS (11 ml), re-pelleted by ultracentrifugation at 110,000×g for 1 h and re-suspended in neuronal medium conditioned by neurons (100 μl). Then, neurons were incubated with 90 μl (high dose) or 10 μl (low dose) of EV suspension, which replaced an equal volume of neuronal medium. At the end of EV treatment, the neurons were incubated with 0.7 μM calcein and 15 μM propidium iodide for 25 min at 37 °C and gently washed with KRH before reading by TECAN Infinite F500 microplate reader at 37 °C in KRH. Neuronal viability was expressed as the ratio of calcein over PI fluorescence. Untreated neurons were used as negative control while neurons exposed to 10 mM glutamate for 2 h 1 day before the assay test were used as positive control for cytotoxicity.

### Statistical analysis

Statistical analysis was performed using the GraphPad Prism 9.0 Software (GraphPad Software Inc., San Diego, CA, USA). One-way ANOVA with Dunnett’s or Bonferroni’s correction for multiple comparisons was used to find differences in the comparison with non-frail CTRL. The Spearman test for correlation between EVs concentration and size and clinical variables was applied. The ability of EV concentration and EV size to discriminate across the groups was also assessed by area under the curve (AUC) obtained by receiver operating characteristic (ROC) curve analyses.

## Results

### Patients’ characteristics

In the present study, 40 patients were enrolled. Of them, 20 were MCI, and the remaining was diagnosed with AD. Moreover, 20 age-matched subjects were included as controls. All patients and CTRLs were subsequently classified in frail and non-frail, according to Canevelli’s Frailty Index (FI) [30]. Clinical characteristics, laboratory details of biomarkers levels and frailty status of the study population are reported in Table 1.

### Characterization of total, neuronal and microglial EVs

Circulating total EVs were isolated from plasma of AD, MCI and CTRL by Exoquick precipitation, followed by affinity capture for the neuronal marker L1CAM or the microglial marker TMEM119 for NDV and MDV purification, respectively. Both total plasma EVs and EVs of neuronal and microglial origin have been characterized by multiple approaches according to the guidelines of the International Society of Extracellular Vesicles (ISEV) [25, 31, 32]. In this study, the purity of EVs was determined by immunoblotting analysis, which showed immunoreactivity of plasma EVs for the EV marker ALIX and negative staining for the non-EV marker GM130, a Golgi marker. NDV-enriched and MDV-enriched fractions were detected with antibodies against L1CAM and TMEM119 respectively. Depleted EV fraction (DEVs) and plasma sample were used as negative and positive controls (Fig. 2).

### Concentration of NDVs and MDVs discriminate non-frail CTRL and MCI

**Fig. 3:**
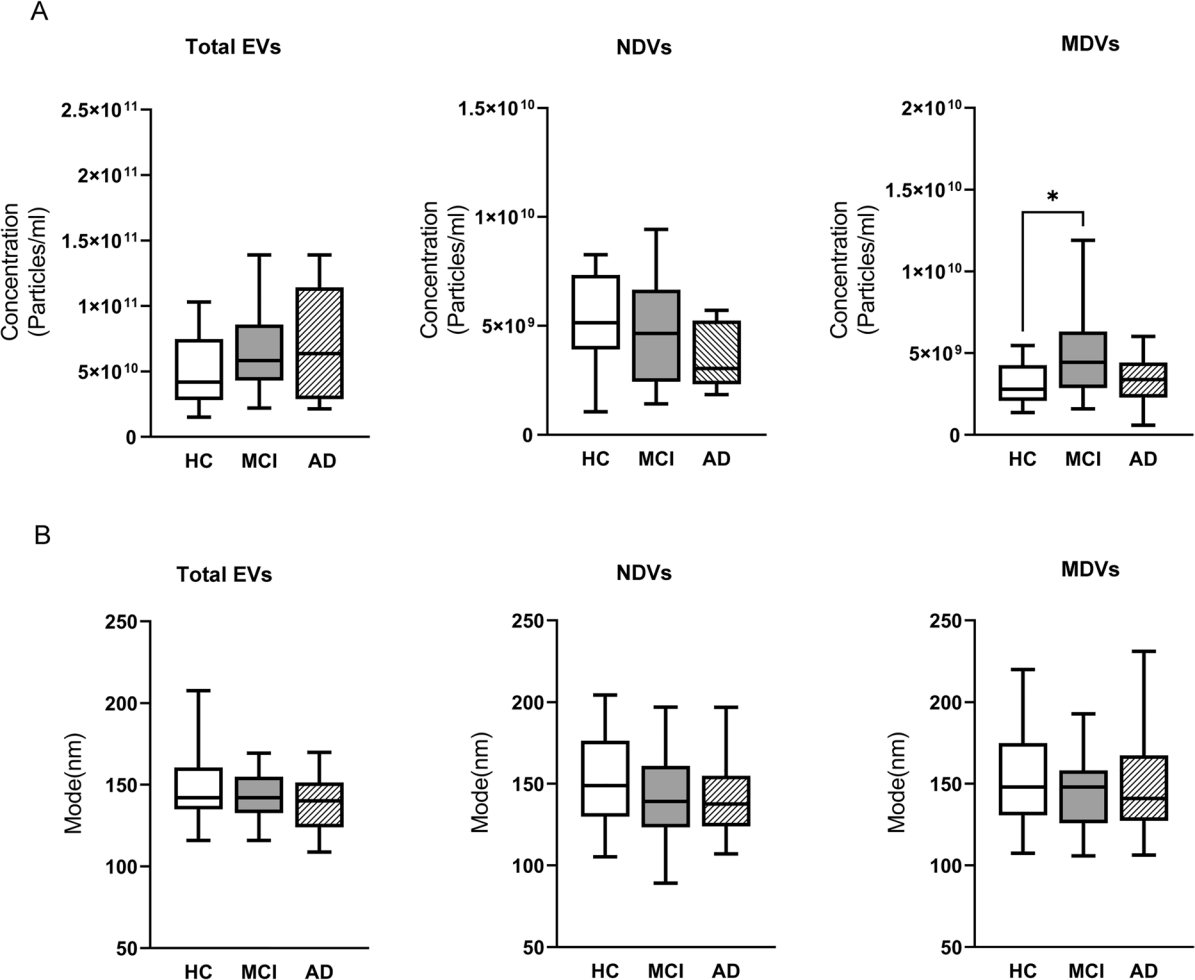
NTA analysis of total EVs, NDVs and MDVs in patients and control subjects. **A** Quantification of EVs concentration (particles/ml) and **B** size (mode, nm) in 20 AD, 20 MCI and 20 CTRL plasma samples. For each box plot, the centre of the line indicates the median and the box limits show 25° and 75° percentile. Bonferroni’s multiple comparison test, *p < 0.05

NTA analysis revealed that the concentration of total plasma EVs and NDVs was similar between patients (AD, MCI) and CTRLs while the concentration of MDVs was significantly higher in the MCI group compared to CTRL (5.38 × 10^9^ ± 3.65 × 10^9^ vs 3.57 × 10^9^ ± 3.21 × 10^9^ particles/ml, *P* = 0.04, Fig. 3A). Total EVs, NDVs and MDVs had similar size distribution, with most circulating EV size ranging between 120.2 and 172 nm in all groups analysed (*P* > 0.05, Fig. 3B).

**Fig. 4:**
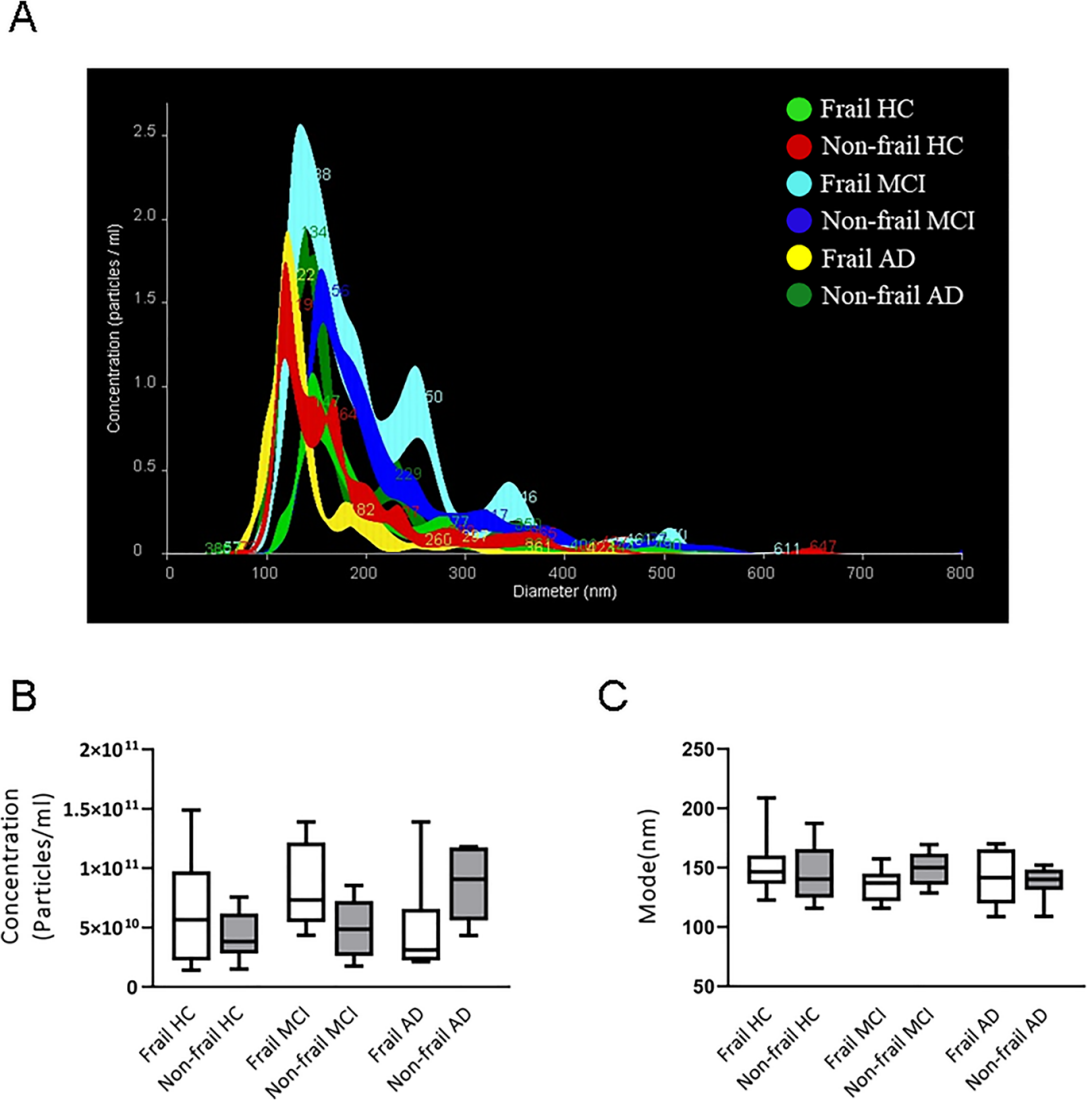
Total EV concentration and size in patients and control subjects according to frailty status. **A** Representative NTA traces of total EVs isolated from plasma of frail or non-frail MCI and CTRL, and AD. **B** NTA analysis of EV concentration (particles/ml) and **C** size (mode, nm) in 10 frail AD, 10 non-frail AD, 10 frail MCI, 10 non-frail MCI, 10 frail CTRL and 10 non-frail CTRL plasma samples. For each box plot, the centre of the line indicates the median and the box limits show 25° and 75° percentile

**Fig. 5:**
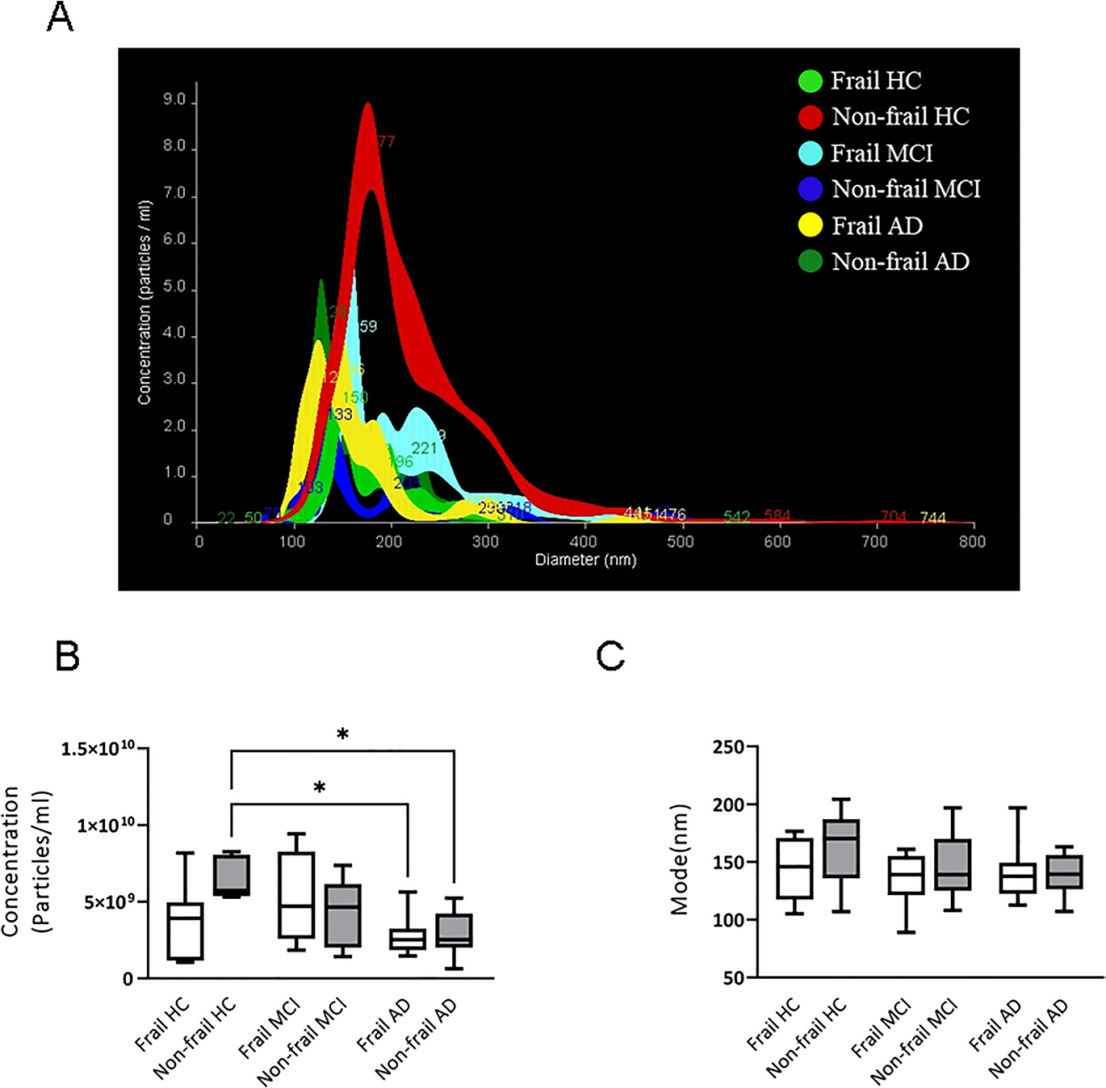
Neuronal-derived vesicle (NDV) concentration and size in patients and control subjects according to frailty status. **A** Representative NTA traces of NDVs isolated from plasma of frail and non-frail AD and frail or non-frail MCI and CTRL. **B** NTA analysis of NDV concentration (particles/ml) and **C** size (mode, nm) in 10 frail AD and 10 non-frail AD, 10 frail MCI, 10 non-frail MCI, 10 frail CTRL and 10 non-frail CTRL plasma samples. For each box plot, the centre of the line indicates the median, and the box limits show 25° and 75° percentile. Dunnett’s multiple comparison test, *p < 0.05

When patients were analyzed according to their frailty status, a trend towards an increase in the number of total vesicles in frail CTRL and frail MCI was observed compared to not frail subjects, albeit differences did not reach the statistical significance (Fig. 4B). Furthermore, no statistically significant differences were observed in vesicle concentration and diameter in frail and non-frail AD patients compared with CTRL (Fig. 4B, C). Evaluation of size and concentration of specific brain-derived EVs revealed that non-frail CTRL had larger and more numerous plasma NDVs than all the other analyzed groups (Fig. 5). Specifically, non-frail CTRL had more NDVs compared to frail and non-AD subjects (7.16 × 10^9^ ± 4.3 × 10^9^ vs 3.65 × 10^9^ ± 1.98 × 10^9^ particles/ml, *P* = 0.019, and 7.16 × 10^9^ ± 4.3 × 10^9^ vs 3.581 × 10^9^ ± 1.98 × 10^9^ particles/ml, *P* = 0.021, Fig. 5A, B; Table 1) as well as frail subjects (with or without MCI), despite the latter differences were not statistically significant. This suggested that production of NDVs might decrease in all conditions of cognitive decline. In addition, NDVs from non-frail CTRL showed a trend towards larger size compared NDVs from the other groups (Fig. 5A, C).

**Fig. 6:**
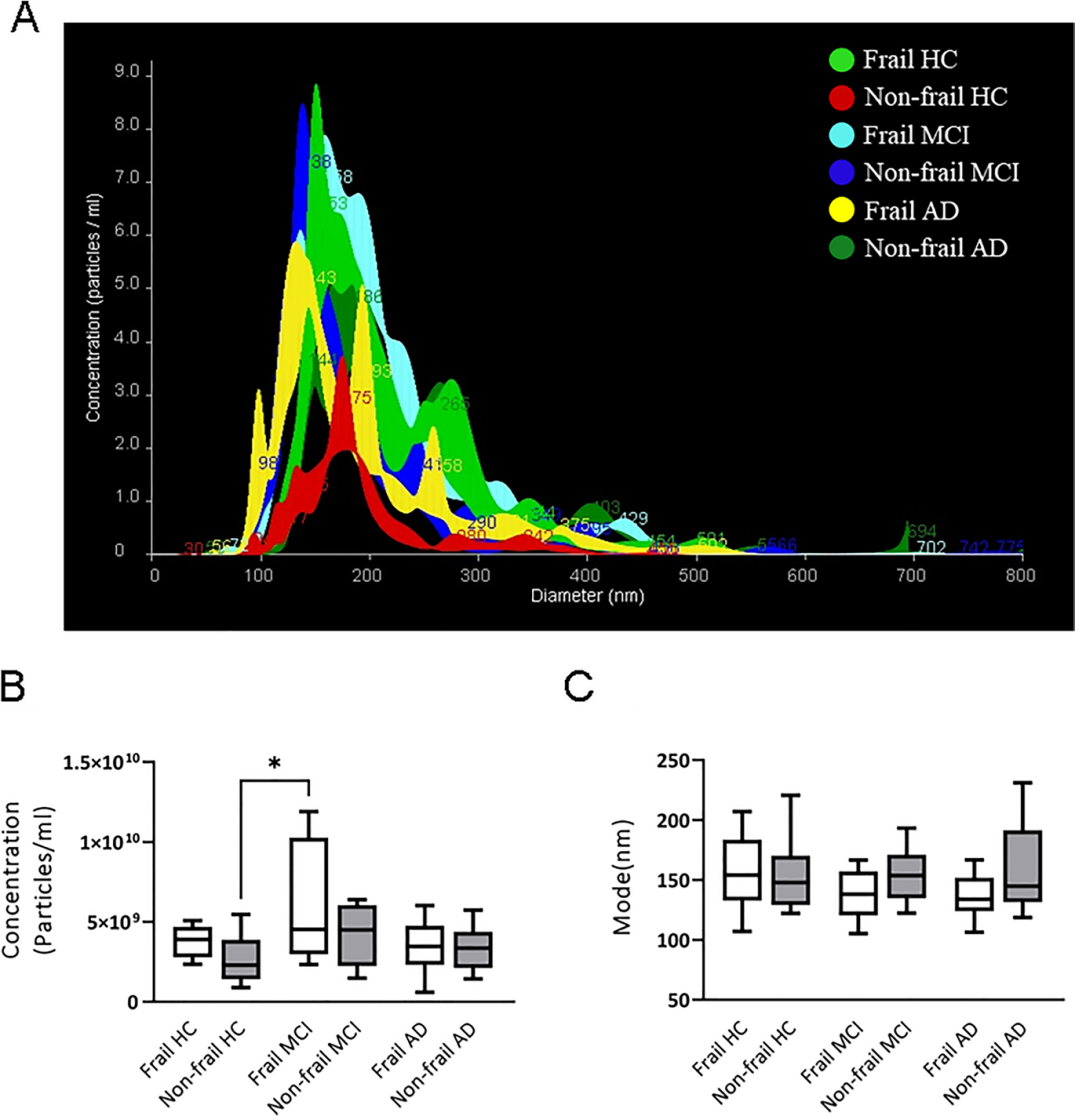
Microglial-derived vesicle (MDV) concentration and size in patients and control subjects according to frailty status. **A** Representative NTA traces of MDVs isolated from plasma of frail and non-frail AD, frail or non-frail MCI and CTRL. **B** NTA analysis of MDV concentration (particles/ml) and **C** size (mode, nm) in 10 frail or non-frail AD, 10 frail or non-frail MCI and 10 frail or non-frail CTRL plasma samples. For each box plot, the centre of the line indicates the median, and the box limits show 25° and 75° percentile. Bonferroni’s multiple comparison test, *p < 0.05

Regarding EVs of microglial origin, NTA data showed a trend to increase concentration of MDVs in frail subjects (with or without MCI) compared to non-frail subjects (Fig. 6), with a significant higher concentration of MDVs in frail MCI patients compared to non-frail CTRL (Fig. 6B, 5.89 × 10^9^ ± 3.98 × 10^9^ vs 3.16 × 10^9^ ±3.04 × 10^9^ particles/ml, *P* < 0.05, Fig. 6A, B; Table 1). Conversely, no significant differences in the analysis of the MDVs size distribution were observed among the study groups (Fig. 6A, C).

### MDVs decrease neuron viability

**Fig. 7:**
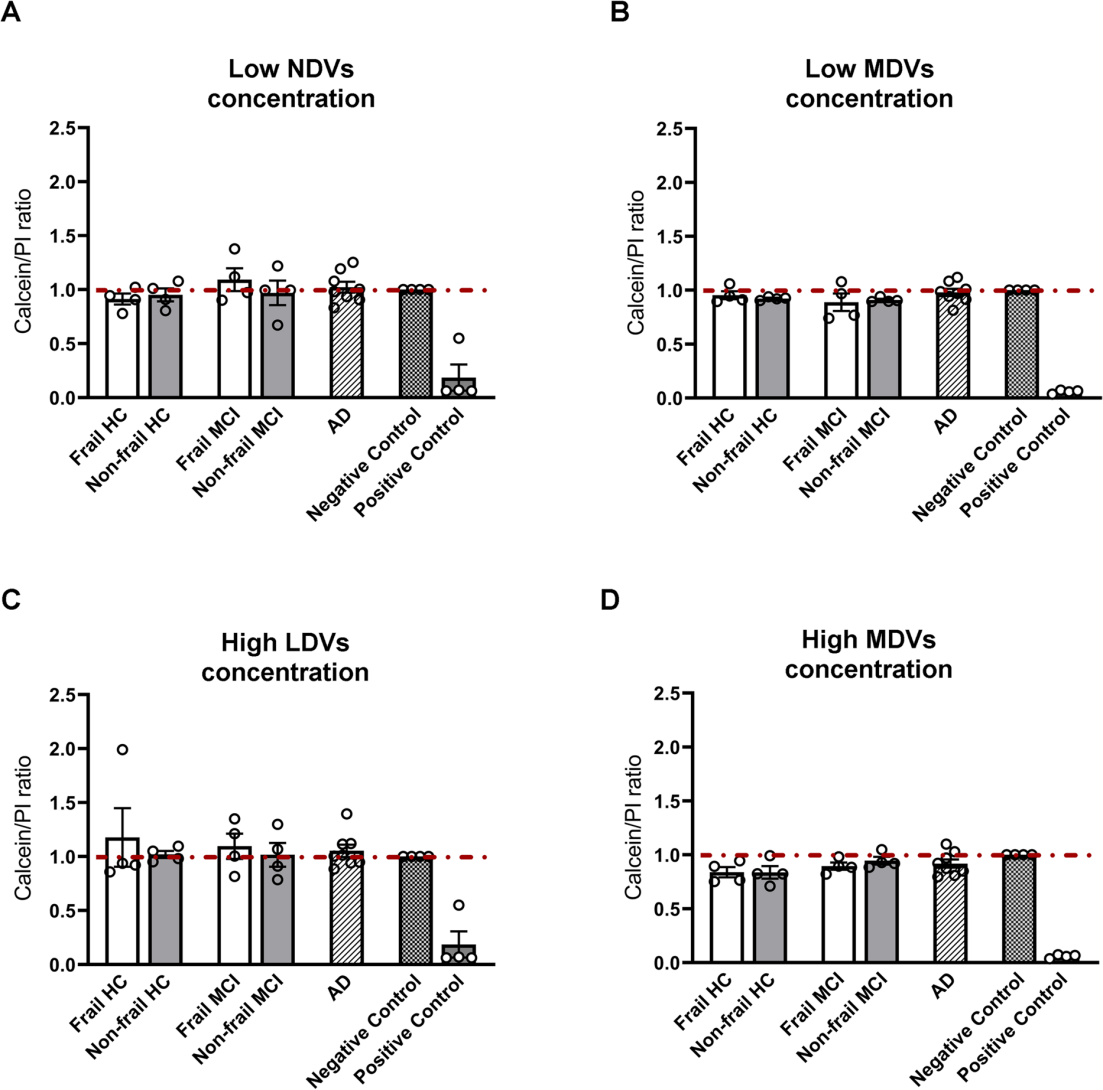
Cytotoxicity assessment of NDVs and MDVs on neuronal cells. Relative fluorescence for calcein-AM/PI of cultured hippocampal neurons exposed to low or high doses of **A**–**C** NDVs and **B**–**D** MDVs isolated from plasma of frail or non-frail AD that are plotted together since no differences were appreciable when considered separated, and frail or non-frail MCI and CTRL. Neurons exposed to glutamate 10 mM or untreated neuronal cultures were used as positive and negative control, respectively. Data are normalized over control and shown as mean ± S.E.M (*n* = 4)

Next, we examined whether NDVs and/or MDVs could affect neuron viability in mouse hippocampal neuron primary cultures. Mature neurons were exposed to two concentrations, a low and a high concentration, of NDVs or MDVs [25], and neuronal viability was assayed by calcein-AM/propidium iodide staining. No significant alterations in neuron viability were observed when neurons were incubated with NDVs (Fig. 7A, C). On the contrary, we found a trend to a generalized neurotoxic effect of MDVs at higher concentration (0.5–1 × 10^9^ particles/ml) (Fig. 7D) whereas only MDVs from frail MCI patients seem to decrease neuron viability at low concentration (0.5–1 × 10^8^ particles/ml, Fig. 7B) although the significance threshold was not reached.

Therefore, not only production of MDVs is higher in frail MCI group, but also MDVs from frail MCI subjects appear to be more toxic to neurons.

### Correlation between EVs characteristics and clinical data

Correlation between total, NDV and MDV concentration, size and clinical parameters relevant to AD diagnosis was considered for analysis in all groups of vesicles and patients. Concentration and size of total plasma EVs did not appear to be correlated with clinical data nor with FI (data not shown). Moreover, no correlations between age and gender and concentration or size distribution of total EVs, NDVs or MDVs were observed in the cohorts investigated.

Conversely, we found both total EV concentration and size are positively correlated with the MDV concentration (*r* = 0.49, *P* = 0.037) and size (*r* = 0.56, *P* = 0.016) in AD group.

### EV concentration discriminate controls from patients

The diagnostic performance of EVs concentration in discriminating across the groups was assessed by a receiver operating characteristic (ROC) curve analysis with Wilson/Brown method. The analysis showed that NDVs concentration is able to discriminate AD patients from non-frail CTRL (AUC = 0.80), with a sensitivity of 78.95% and a specificity of 85.7%, considering the cut-off of 5.27 × 10^9^ particles/ml. Moreover, it has been revealed that microglial EV concentration discriminates frail MCI vs non-frail CTRL (AUC = 0.76) with a sensitivity of 80% and a specificity of 70%, considering the cut-off of 2.69 × 10^9^ (Figure S1). Finally, to test the diagnostic performance of EV concentration and size parameters, we performed the ROC analysis by using the EV concentration/EV size ratio to distinguish between patients and non-frail CTRL. The analyses showed that concentration/size ratio of total plasma EVs was able to discriminate between frail MCI and non-frail CTRL, with a sensitivity 80% of and a specificity of 70% (with a cut-off point of 3.9 × 10^8^) (Figure S2).

## Discussion

Over the last years, EVs have emerged to exert a pivotal role in neurodegenerative diseases, including AD [23, 33]. Moreover, the development of methods allowing the isolation of brain-cell–derived EVs from human plasma opened interesting chances in exploiting them as a source of biomarkers for diagnosis and prediction [7, 34–37].

In the present study, we aimed to examine the changes in EV concentration in the context of frailty and dementia and, moreover, for the first time, we investigated NDVs and MDVs in plasma obtained from frail and non-frail subjects.

For the isolation of plasma brain–derived vesicles, specific markers have been used. In particular, for NDVs, the L1CAM marker was chosen according to recent literature that widely accepts its use as marker for neuron-derived EVs [7, 32, 38]. Considering microglia, there are several markers eligible, and they include surface intracellular and released molecules. The most widely used are ionized calcium-binding- adapter molecule 1 (IBA-1; [39]), cluster of differentiation receptors as CD11b [40] and other molecules (see for review [41, 42]). In particular, CD11b expression at the EV surface might reflect in principle the activation state of donor microglia, but it is not able to discriminate between EVs produced by microglia from those released by other myeloid cells. However, to the current knowledge, the most specific general microglia marker is transmembrane protein 119 (TMEM119) [41, 43].

The key finding of this study is the demonstration that the MDVs are increased in number in the plasma of MCI participants with frailty, a condition that likely contributes to the relevant variability of health outcome in elderly and that might affect or accelerate the degenerative processes. Specifically, we found that MDVs appear to damage cultured neurons. Notably, we showed that MDV concentration is particularly increased in frail MCI patients compared to non-frail CTRL with no difference in their size, suggesting that MDV release is associated to the frailty condition and that frailty exacerbates the release of MDVs in MCI.

EV production from microglia is known to increase upon microglia activation in several pathological contexts, especially under sustained activation, when microglia undergo phenotypic specification into neurodegenerative or disease-associated microglia (MGnd/DAM) [44–46], which play a pivotal role in disease progression [47]. Several evidences support the key role of microglia dysfunction in AD pathogenesis.

Microglia activation in AD is a dynamic and complicated process that spans from progressive activation to partial and full activation depending on the context and the type or extent of the CNS damage. In the early stage of the disease, microglial activation drives Aβ phagocytose to inhibit Aβ deposition, exerting in this way neurotrophic effects. With the progression of the disease, chronically activated microglia gradually lose their ability or efficiency to eliminate Aβ, whereas they continuously produce pro-inflammatory and neurotoxic factors as well as reactivate aberrant synaptic pruning. All these processes at the end turn to neurodegeneration [48]. In line with this, recent evidence showed that microglia is activated in the prodromal MCI phase [49], and elevated concentration of microglial EVs, positive for the myeloid marker IB4, was previously detected by flow cytometry in the CSF of MCI patients [26, 50]. Importantly, recent studies also clarified that EVs released by DAM contribute to neurodegeneration, by favoring the propagation of structural and functional synaptic dysfunctions via dissemination of Aβ and tau misfolded proteins [47, 51–53].

Consistent with these findings, we also observed that in the overt pathological condition, MDVs appear to be the most represented as shown with the positive correlation found among concertation and size of total EVs and MDVs in AD patients. Moreover, we observed that MDVs from frail MCI appear to exert toxic effects on neurons, likely decreasing cell viability. This evidence, together with elevated MDVs production in MCI patients (also observed in [26]), supports a pathogenic contribution of MDVs to neuron and axon damage, which may underlie cognitive impairment and possible progression to dementia.

In our study, despite the total number of circulating EVs was unchanged, we show that NDVs were significantly decreased in AD group and displayed a trend to decrease in the MCI group compared to non-frail CTRL, suggesting that circulating NDVs may be a more powerful biomarker for AD compared to total EVs.

Of note, we found that NDV concentration was able to distinguish between AD group and non-frail CTRL with a sensitivity of 78.95% and a specificity of 85.7%.

The significant decrease in NDVs concentration with progression of cognitive decline supports the hypothesis that NDVs may track cognitive decline in AD, as recently proposed [38]. Reduced levels of NDVs may reflect loss of neurons and/or altered neuronal homeostasis in AD [54, 55]. Importantly, such alterations may also affect the cargo of NDVs, not only the number of vesicles. This has been shown by a recent study that identified in NDVs two proteins, complement component 7 (C7) and Zyxin, as novel marker of transition from normal aging to MCI and then to clinically defined AD [56].

To the best of our knowledge, there are only two previous studies in which total plasma EVs concentration was measured in the elderly in association with frailty. Results showed no correlation between frailty status and EV concentration in circulation [57, 58], as we confirmed in our study. In addition, opposite to our results, previous evidence underlined a significant decrease in total circulating plasma EVs in AD patients compared to controls [23]. Differences in the experimental conditions and lack of standardized procedures might justify this discrepancy.

However, the diagnostic performance of combined total EVs size and concentration is high and able to distinguish between frail MCI and non-frail CTRL. These data are consistent with previous studies describing altered total EV concentration in AD and frontotemporal dementia [23, 59]. Nevertheless, the reasons underlying these changes in EV dosage remain to be clarified.

A recent cross-sectional analysis of data performed in MCI and AD evaluated the contribution of frailty with the cognitive changes and the pathological AD modifications. Here, the authors debated that frailty moderates the relationship with cognitive changes and dementia [60]. In agreement to these observations, our results could explain the biological mechanism behind such evidence, due to the highest level of MDVs in frail MCI, with consequent greater neuronal toxicity. To sum up, the increased number of MDVs found in plasma of frail MCI might have some consequences in the neighbouring neuronal cells, since it is well established the involvement of MDVs in inducing neurite outgrowth, modulating neuronal activity [61]. The MDV neurotoxicity found in frail MCI confirmed this hypothesis and further contribute to shed light to the pathogenic mechanisms occurring in MCI in evolving to dementia. This result, together with the fact that MDVs were even more numerous in this own group of subjects, might lead us to hypothesize their involvement in spreading cognitive decline.

A limitation of this study is represented by the lack of availability of follow-up of MCI patients representing an actual limitation to verify the real influence of the higher concentration of MDVs in frail MCI subjects on the progression of cognitive impairment to dementia.

The current study indicates that neuronal and microglial EV production changes during cognitive decline, as a possible adaptive mechanism in neurodegenerative processes characteristic of MCI and dementia and provides insights into changes in size and concentration of plasma EVs in relation to the frailty status: we found the decrease in NDVs release as a common alteration in cognitive decline condition, whereas MDV dosage might be a promising marker of progression for frail MCI patients.

Although NDVs and MDVs size and concentration may reflect altered mechanisms leading to cognitive impairment and frailty in elderly, further analysis are required to comprehensively shed light on the roles of these specific brain-derived vesicles in the context of frailty.

## Supplementary information

Figure S1: ROC curves identifying the best cutoff for each EVs types, discriminating all patients from non-frail CTRL. (A) In total EVs, AUC non-frail CTRL vs frail CTRL 0.62 (green); AUC non-frail CTRL vs non-frail MCI 0.57 (blue); AUC non-frail CTRL vs frail MCI 0.77 (red); AUC non-frail CTRL vs AD 0.64 (black). (B) In neuronal EVs, AUC non-frail CTRL vs frail CTRL 0.76 (green); AUC non-frail CTRL vs non-frail MCI 0.68 (blue); AUC non-frail CTRL vs frail MCI 0.60 (red); AUC non-frail CTRL vs AD 0.80 (black). (C) In microglial EVs, AUC non-frail CTRL vs frail CTRL 0.68 (green); AUC non-frail CTRL vs non-frail MCI 0.70 (blue); AUC non-frail CTRL vs frail MCI 0.76 (red); AUC non-frail CTRL vs AD 0.63 (black). AUC comparison was performed with Wilson/Brown method.

Figure S2: The ratio of EVs concentration/ size was used to evaluate the diagnostic capacity to discriminate all patients from non-frail CTRL. (A) In total EVs, AUC non-frail CTRL vs frail CTRL 0.62 (green); AUC non-frail CTRL vs non-frail MCI 0.52 (blue); AUC non-frail CTRL vs frail MCI 0.81 (red); AUC non-frail CTRL vs AD 0.64 (black). (B) In neuronal EVs, AUC non-frail CTRL vs frail CTRL 0.65 (green); AUC non-frail CTRL vs non-frail MCI 0.63 (blue); AUC non-frail CTRL vs frail MCI 0.61 (red); AUC non-frail CTRL vs AD 0.77 (black). (C) In microglial EVs, AUC non-frail CTRL vs frail CTRL 0.69 (green); AUC non-frail CTRL vs non-frail MCI 0.69 (blue); AUC non-frail CTRL vs frail MCI 0.78 (red); AUC non-frail CTRL vs AD 0.61 (black). AUC comparison was performed with Wilson/Brown method.
