# Supplementary figures and images for: Plasma microglial-derived extracellular vesicles are increased in frail patients with Mild Cognitive Impairment and exert a neurotoxic effect
Source: GeroScience. 2023 Feb 1;45(3):1557–71. doi: 10.1007/s11357-023-00746-0 (PMC10400496; doi:10.1007/s11357-023-00746-0)

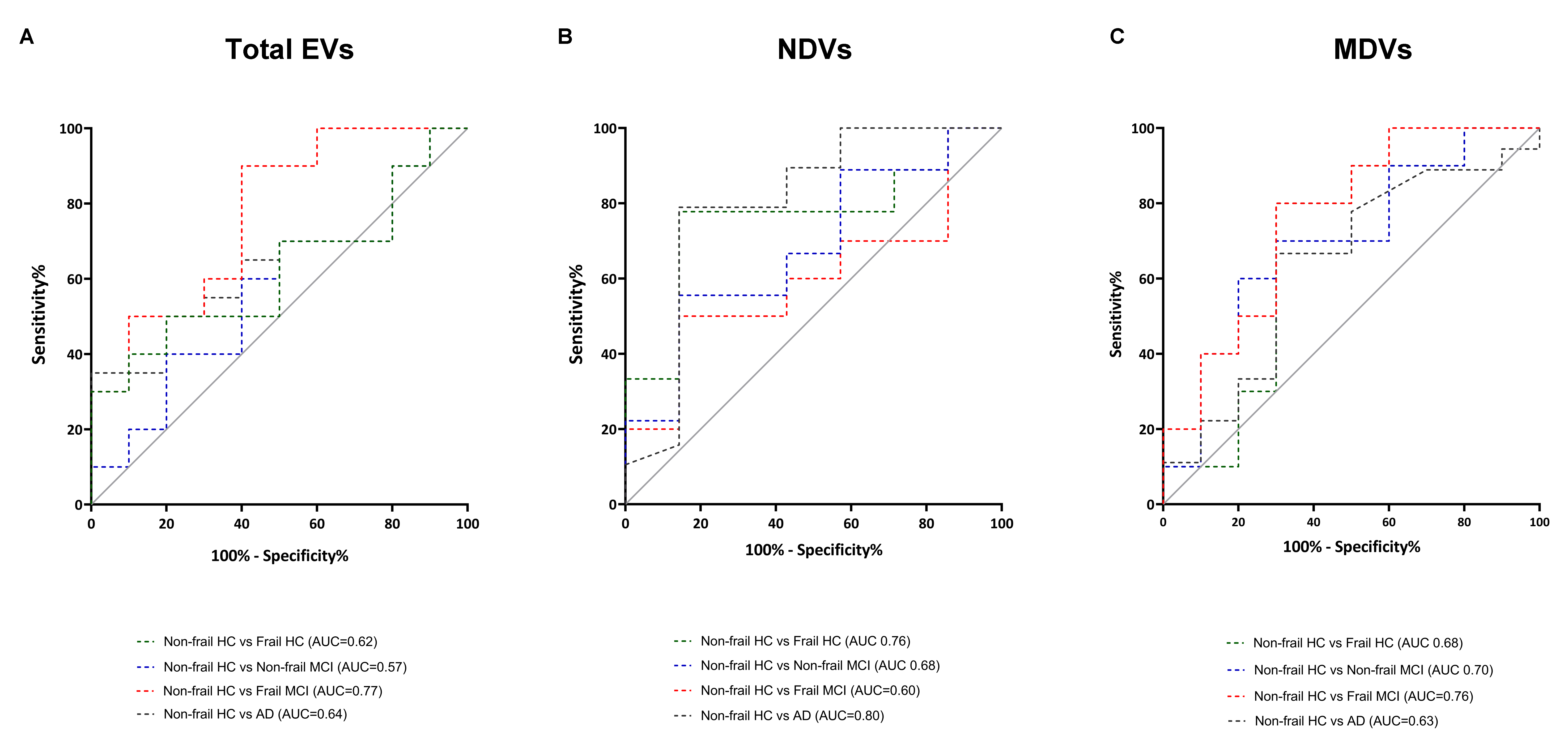

Supplement: Supplementary file 1 — ROC curves identifying the best cutoff for each EVs types, discriminating all patients from non-frail CTRL. (A) In total EVs, AUC non-frail CTRL vs frail CTRL 0.62 (green); AUC non-frail CTRL vs non-frail MCI 0.57 (blue); AUC non-frail CTRL vs frail MCI 0.77 (red); AUC non-frail CTRL vs AD 0.64 (black). (B) In neuronal EVs, AUC non-frail CTRL vs frail CTRL 0.76 (green); AUC non-frail CTRL vs non-frail MCI 0.68 (blue); AUC non-frail CTRL vs frail MCI 0.60 (red); AUC non-frail CTRL vs AD 0.80 (black). (C) In microglial EVs, AUC non-frail CTRL vs frail CTRL 0.68 (green); AUC non-frail CTRL vs non-frail MCI 0.70 (blue); AUC non-frail CTRL vs frail MCI 0.76 (red); AUC non-frail CTRL vs AD 0.63 (black). AUC comparison was performed with Wilson/Brown method. [file 11357_2023_746_MOESM1_ESM.tif]

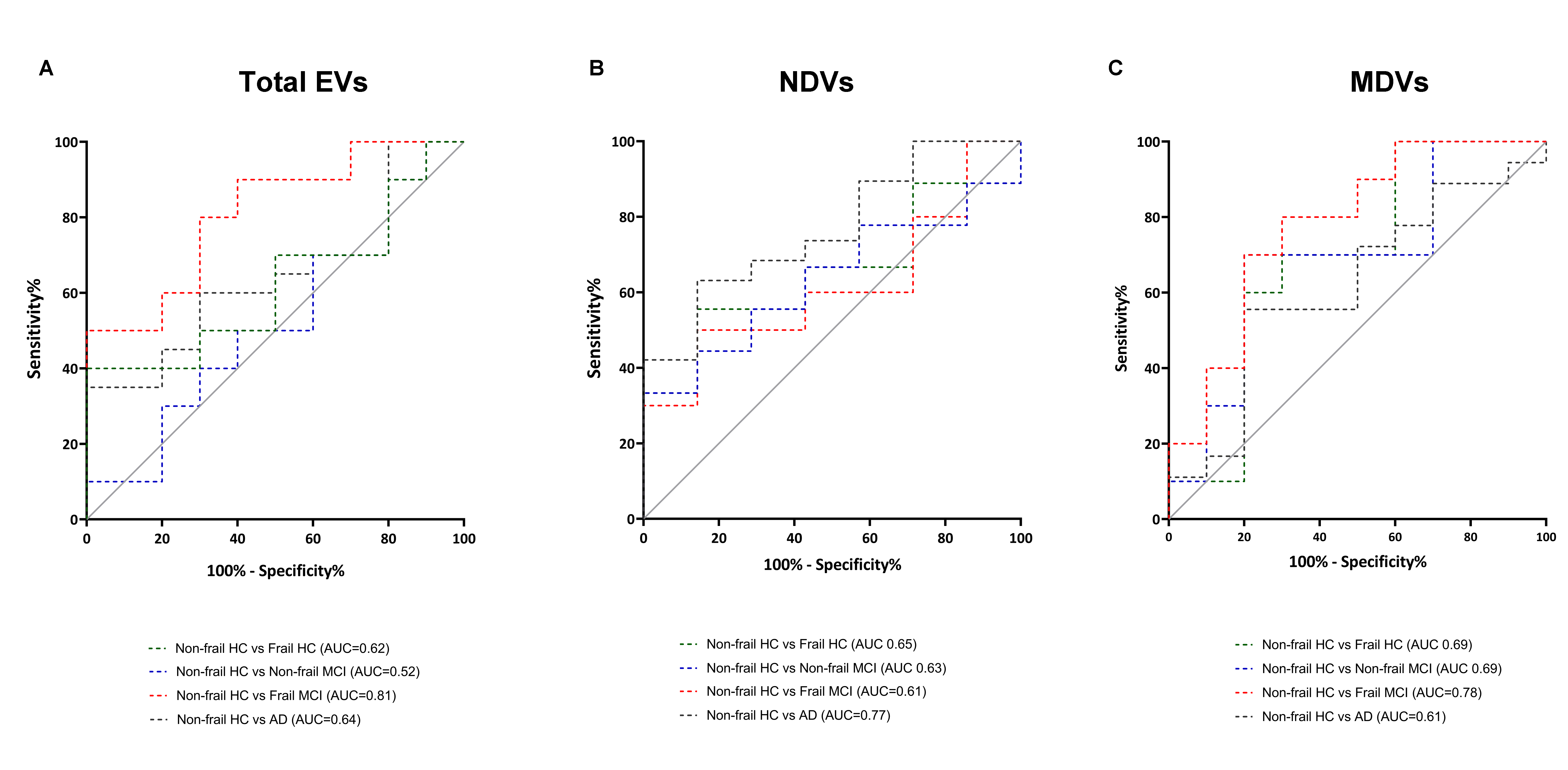

Supplement: Supplementary file 2 — The ratio of EVs concentration/ size was used to evaluate the diagnostic capacity to discriminate all patients from non-frail CTRL. (A) In total EVs, AUC non-frail CTRL vs frail CTRL 0.62 (green); AUC non-frail CTRL vs non-frail MCI 0.52 (blue); AUC non-frail CTRL vs frail MCI 0.81 (red); AUC non-frail CTRL vs AD 0.64 (black). (B) In neuronal EVs, AUC non-frail CTRL vs frail CTRL 0.65 (green); AUC non-frail CTRL vs non-frail MCI 0.63 (blue); AUC non-frail CTRL vs frail MCI 0.61 (red); AUC non-frail CTRL vs AD 0.77 (black). (C) In microglial EVs, AUC non-frail CTRL vs frail CTRL 0.69 (green); AUC non-frail CTRL vs non-frail MCI 0.69 (blue); AUC non-frail CTRL vs frail MCI 0.78 (red); AUC non-frail CTRL vs AD 0.61 (black). AUC comparison was performed with Wilson/Brown method. [file 11357_2023_746_MOESM2_ESM.tif]
